# Growth Kinetics and Mechanistic Action of Reactive Oxygen Species Released by Silver Nanoparticles from *Aspergillus niger* on *Escherichia coli*

**DOI:** 10.1155/2014/753419

**Published:** 2014-06-16

**Authors:** Shivaraj Ninganagouda, Vandana Rathod, Dattu Singh, Jyoti Hiremath, Ashish Kumar Singh, Jasmine Mathew, Manzoor ul-Haq

**Affiliations:** Department of Microbiology, Gulbarga University, Gulbarga, Karnataka 585106, India

## Abstract

Silver Nanoparticles (AgNPs), the real silver bullet, are known to have good antibacterial properties against pathogenic microorganisms. In the present study AgNPs were prepared from extracellular filtrate of *Aspergillus niger*. Characterization of AgNPs by UV-Vis spectrum reveals specific surface plasmon resonance at peak 416 nm; TEM photographs revealed the size of the AgNPs to be 20–55 nm. Average diameter of the produced AgNPs was found to be 73 nm with a zeta potential that was −24 mV using Malvern Zetasizer. SEM micrographs showed AgNPs to be spherical with smooth morphology. EDS revealed the presence of pure metallic AgNPs along with carbon and oxygen signatures. Of the different concentrations (0, 2.5, 5, 10, and 15 *μ*g/mL) used 10 *μ*g/mL were sufficient to inhibit 10^7^ CFU/mL of *E. coli*. ROS production was measured using DCFH-DA method and the the free radical generation effect of AgNPs on bacterial growth inhibition was investigated by ESR spectroscopy. This paper not only deals with the damage inflicted on microorganisms by AgNPs but also induces cell death through the production of ROS released by AgNPs and also growth kinetics of *E. coli* supplemented with AgNPs produced by *A. niger*.

## 1. Introduction

The broad spectrum antimicrobial properties of silver nanoparticles (AgNPs) encourage its use in biomedical applications; with the rapid development of nanobiotechnology, applications have been extended further and now silver is the engineered nanomaterial most commonly used in consumer products [1, 2]. It has been known that silver and its compounds have strong inhibitory and bactericidal effects as well as broad spectrum of antimicrobial activities for bacteria [3–5], fungi [6], and viruses [7]. Compared with other metals, silver exhibits higher toxicity to microorganisms while it exhibits lower toxicity to mammalian cells [8]. AgNPs had been known for a long time but have been paid little attention. Advances in recent research on nanobiotechnology appear to revive the potential of AgNPs for antimicrobial applications [9].

Nanoparticles are used in many applications as they exhibit interesting and useful properties, which may be very different to their parent bulk material, because nanoparticles have extremely high surfaced area to volume ratios, so the properties of the nanoparticles are dominated by surface atom contribution [10]. Biologically inspired nanoparticle syntheses are currently a rapid expanding area of research which draws on many different disciplines. Recently, there has been lots of interests in biologically controlled synthesis as a greener, cheaper alternative to other methods [11].

The use of fungi in the synthesis of AgNPs is a relatively recent addition and holds promise for large scale nanoparticles production. In fact, fungi secrete large amount of the enzymes involved in AgNPs synthesis and are simpler to grow both in laboratory and at industrial scale [12].

In the present study we emphasis on biosynthesis of AgNPs from extracellular filtrate of* Aspergillus niger* and study the mechanistic action of reactive oxygen species (ROS) generated by AgNPs through electron spin resonance spectroscopy (ESR) from the surface of Ag^+^ on pathogenic* Escherichia coli* and confirm the obtained transmission electron microscopy (TEM) results by Kinetic studies through growth curve.

## 2. Materials and Methods

### 2.1. Biosynthesis and Characterization of AgNPs

The fungus* A. niger* was grown in 250 mL Erlenmeyer flasks containing malt extract, glucose, yeast extract, and peptone (MGYP- 0.3%, 1%, 0.3%, and 0.5% and pH 6) at temperature 29°C. After incubation mycelium was separated by filtration, washed with sterile distilled water to remove traces of media contents, and resuspended in 100 mL distilled water for about 48 hrs. The suspension was filtered through Whatman filter paper number 1. The cell filtrate was challenged with silver nitrate (1 mM) for AgNPs biosynthesis.

The Optical density of the synthesized AgNPs was characterized by UV-Vis spectroscopy (T 90+ UV-VIS spectrophotometer), the interaction between protein and AgNPs was evaluated using Fourier-transform infrared spectroscopy (FTIR) (Perkin Elmer model 783 spectrophotometer), the size and morphology of the AgNPs were examined by transmission electron microscopy (TEM) (Hitachi H 7500 ID, Japan), the particle size distribution of AgNPs was evaluated using Malvern Zetasizer nanoseries spectrometer (Malvern Instruments Ltd.), shape and surface morphology of nanoparticles was characterized by using scanning electron microscopy (SEM) (JEOL Model JSM-6390 LV), the presence of elemental silver was confirmed through energy dispersive spectroscopy (EDS) (JEOL Model JED-2300), and release of reactive oxygen species (ROS) from the surface of AgNPs was evaluated by employing electron spin resonance spectroscopy (ESR) (JES-FA 200****ESR Spectrometer, JEOL Japan).

### 2.2. Bactericidal Activity of AgNPs

Agar dilution method was used to study the bactericidal activity of AgNPs on* E. coli*. Nutrient Agar (NA) supplemented with various concentrations of AgNPs (0, 2.5, 5, 10, and 15 *μ*g/mL) and each plate was inoculated with 10^7^ CFU/mL of* E. coli* by spread plating. Plates inoculated with* E. coli* but without AgNPs were used as control. Number of surviving bacteria in agar plates was counted after 24 hours of incubation at 37°C [13, 14].

### 2.3. Bacterial Growth Kinetics against Different Concentrations of AgNPs

To study the bacterial growth curve,* E. coli* culture was inoculated with fresh colonies and incubated for 12 hour overnight at 37°C in nutrient broth (NB). Bacterial growth curves were determined by measuring the optical density (OD) at 600 nm using a spectrophotometer. At this the juncture the OD obtained was 1.0. To different concentrations (0–15 *μ*g/mL) of AgNPs the aforesaid* E. coli* culture was added and the turbidity was measured at different time intervals (0, 5, 10, 15, 20, and 25 hrs) [14]. Experiment was repeated thrice.

### 2.4. Release of ROS from the Surface of AgNPs and Its Mechanism

The interaction of AgNPs with* E. coli* was assessed using TEM. Broth containing* E. coli* exposed to AgNPs was subjected to TEM at a time intervals of 1, 5, 8, and 12 hrs, respectively. The samples which were subjected to TEM studies were also sent for ESR studies to detect the free radical generation from the surface of silver. ESR is an analytical method to detect the free radical generated from the surface of silver. It is based on absorption of microwave radiation by an unpaired electron when it is exposed to a strong magnetic field. AgNPs that contain free radicals therefore are detected by ESR. Free radical generation from the surface of Ag was recorded using ESR spectrophotometer. The generation of ROS from AgNPs was measured using 2′,7′-dichlorofluorescein diacetate (DCFH-DA) [14]. 2′,7′-Dichlorodihydrofluorescein diacetate (DCFH-DA) is one of the most widely used techniques for directly measuring the redox state of a cell. DCFH-DA, a cell permeable, nonfluorescent precursor of DCF can be used as an intracellular probe for oxidative stress. It has many advantages over other techniques developed as it is very easy to use, extremely sensitive to changes in the redox state of a cell, inexpensive, and can be used to follow changes in ROS over time. 10^7^ CFU/mL of cells were treated with 20 *μ*g/mL of AgNPs and incubated at 37°C for 5 h then centrifuged at 4°C for 15 min at 600 ×g and the obtained supernatant was treated with 100 *μ*M DCFH-DA for 1 h. The ROS formed was measured using Fluorescence spectrometry.

To check the inhibition of* E. coli* targeted through the ROS produced by AgNPs from* A. niger*, we conducted a separate experiment using ascorbic acid as an antioxidant which acts as a scavenger. NA plates supplemented with AgNPs (10 *μ*g/mL) were also incorporated with 10 mM ascorbic acid as a scavenger. Thus the plates were inoculated with fresh 12 hrs cultures of* E. coli* and the surviving rate was counted after 24 hrs of incubation [15–17].

## 3. Results and Discussion

### 3.1. Biosynthesis and Characterization of AgNPs

**Figure 1:**
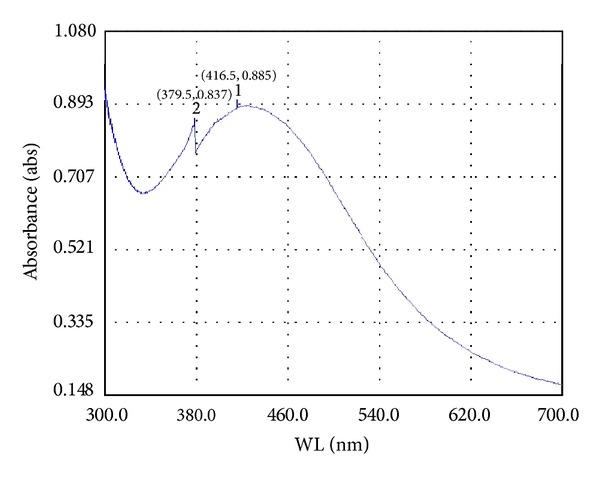
UV-Vis-spectrum of silver nanoparticles.

The production of AgNPs by* A. niger* was indicated by the appearance of brown color in the reaction mixture. The UV-Vis spectrum for AgNPs is obtained by exposing the sample to UV-light from a light source. The specific surface plasmon resonance is responsible for their unique remarkable optical phenomenon. A single peak with a maximum of 416 nm corresponding to the surface plasmon resonance of AgNPs was observed in the UV-Vis spectrum (Figure 1). Singh et al. [3] reported the extracellular biosynthesis of AgNPs using endophytic fungus* Penicillium* sp. at the maximum absorbance peak of 425 nm and also reported that increase in concentration of silver nitrate increases the particle size due to aggregation of larger AgNPs. In an another study Sondi and Salopek-Sondi [13] reported the surface Plasmon band at 405 nm; at this peak the appearance of brown color clearly indicates the formation of AgNPs.

**Figure 2:**
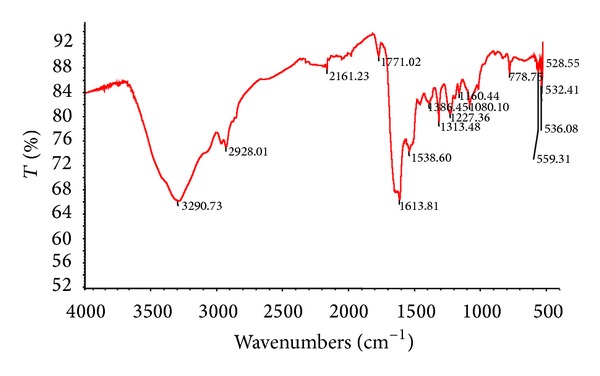
FTIR spectra of silver nanoparticles from* A. niger.*

The interaction between protein and AgNPs was analyzed by Fourier-transform infrared spectroscopy (FTIR). The AgNPs suspension was centrifuged at 10, 000 rpm/10 min and dried sample analysis was recorded on Perkin Elmer one IR spectrophotometer in the range from 450 to 3000 cm^−1^ (Figure 2). The representative spectra in the region of 3000 to 450 cm^−1^ revealed the presence of different functional groups like 3290.73**—**secondary amide (N–H stretch, H-bonded), 2928.01**—**alkane (C–H stretching), 2161.23**—**alkyne (C*≡*C stretching), 1771.02**—**anhydride (C=O stretching), 1613.81**—**alkene (C=C stretching), 1538.60**—**aromatic (C–C stretching), 1386.45, 1313.48**—** and 1080.10**—**primary alcohol (C–O stretching) and 528.55**—**alkene (=C–H bending), respectively. Rathod et al. [6] reported that proteins present in the extract of fungus* Rhizopus stolonifer* can bind to the AgNPs through either free amino or carboxyl groups in the proteins and also they reported different functional groups absorbing characteristic frequencies of FTIR radiation. And also our result correlates with Parashar et al. [17] who reported the possible reaction mechanism for the reduction of Ag^+^ using Guara (*Psidium guajara*) leaf extract. Thus, FTIR is an important and popular tool for structural elucidation and compound identification.

**Figure 3:**
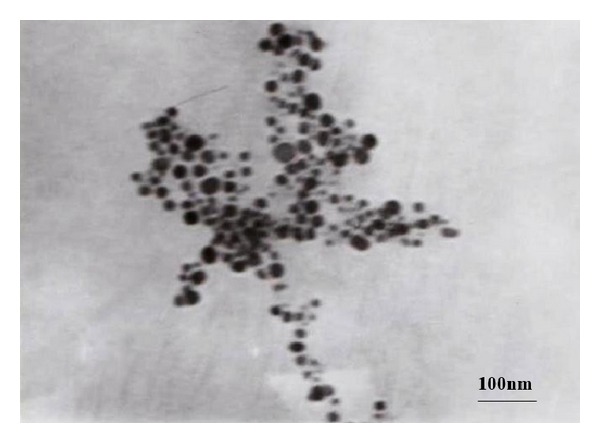
TEM micrograph of silver nanoparticles synthesized using extracellular filtrate of* A. niger.*

A drop of AgNPs solution was placed on the carbon coated copper grids and kept under vacuum before loading them onto a specimen holder. Then TEM micrographs were taken by prepared grids to determine the size and shape of the produced AgNPs.  Figure 3 revealed the particles are spherical in shape and size of the particles is between 20 and 55 nm. Rathod et al. [6] synthesized AgNPs from* Rhizopus stolonifer* and reported that the size is ranging between 5 and 50 nm suggesting that biological molecules could possibly perform the function for the stabilization of the AgNPs and also reported that the AgNPs synthesized by this route are fairly stable even after prolonged storage. Size and shape of AgNPs as revealed by Kora and Arunachalam [15] were 45 nm.

**Figure 4:**
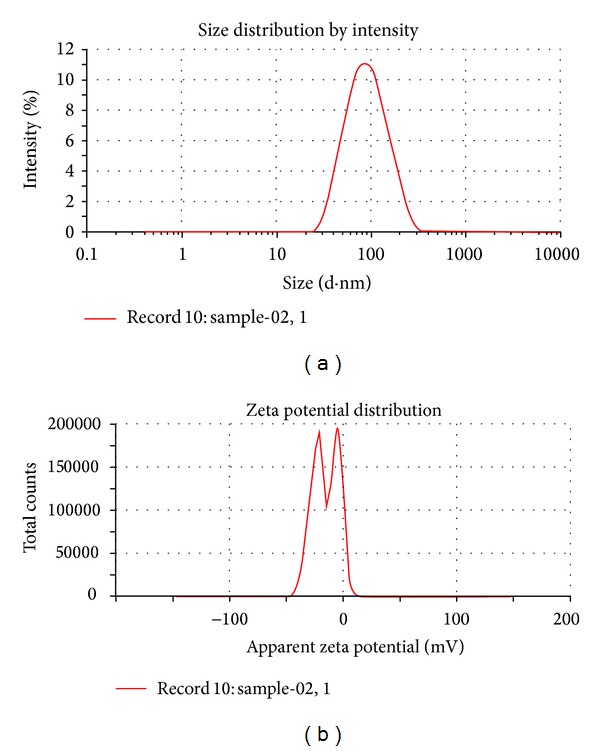
(a) Particle size distribution of AgNPs by intensity with Zeta analyzer, (b) Zeta potential.

The particle size distribution of the AgNPs was shown under different categories like size distribution by volume and by intensity. The average diameter of the particles was found to be 73 nm (100% intensity) (Figure 4(a)) with a zeta potential that was −24 mV (Figure 4(b)). The synthesized AgNps are well distributed with respect to volume and intensity indicates the well dispersion of AgNPs. The average diameter of the particles was found to be 127, 100% intensity and width was found to be 37.25 nm showing monodispersity of the particles using the extract of the plant* Foeniculum vulgare*, as reported by Bonde [18].

**Figure 5:**
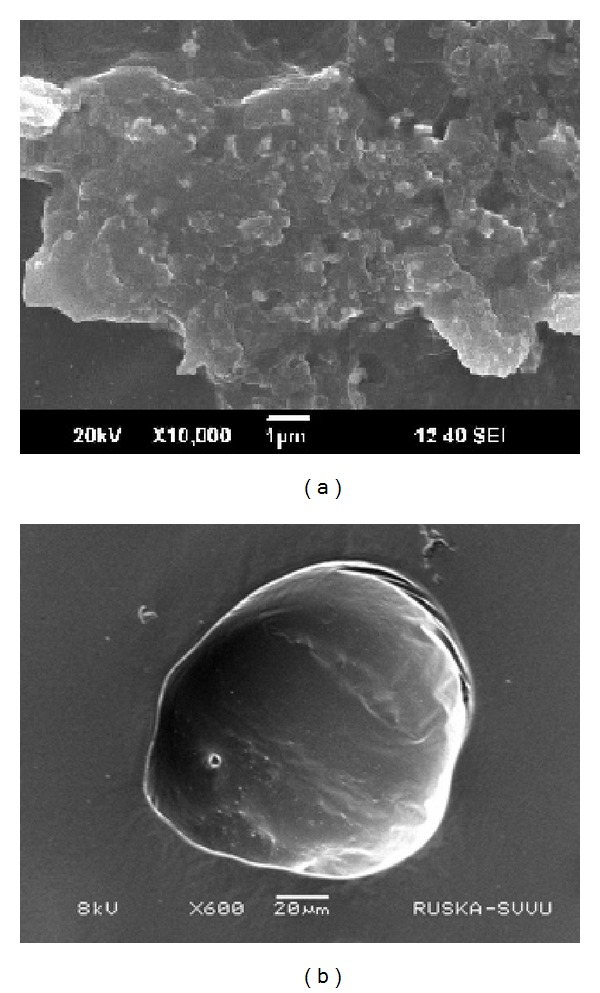
(a) SEM micrograph of silver nanoparticles, (b) single nanoparticle with spherical and smooth morphology.

Scanning electron microscopy (SEM) was used to record the photomicrograph images of synthesized AgNPs. A small volume of AgNPs suspension was taken for SEM analysis on electromicroscope stub. The stubs were placed briefly in a drier and then coated with gold in an ion sputter. Pictures were taken by random scanning of the stub. Shape and surface morphology of AgNPs were studied by SEM.  Figure 5(a) shows the distribution of AgNPs. SEM micrograph of single nanoparticle reveals the spherical and smooth morphology of the nanoparticle (Figure 5(b)).

**Figure 6:**
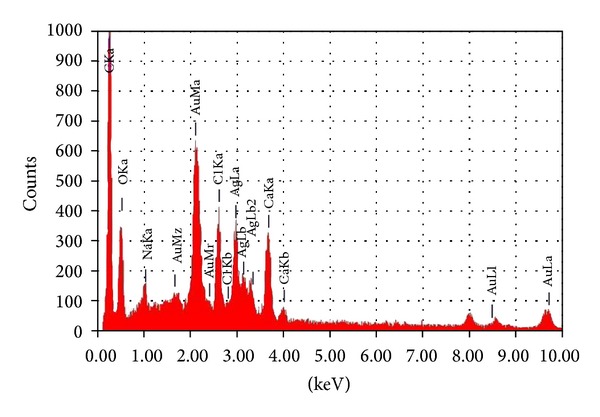
EDS spectrum of extracellular synthesized silver nanoparticles.

Energy dispersive spectroscopy (EDS) samples were prepared on a copper substrate by drop coating of AgNPs. The elemental analysis was examined by EDS. The presence of an optical absorption band at ~3 eV reveals the presence of pure metallic AgNPs along with the C and O signatures that might be from the stabilizing protein (Figure 5). As our result correlates with Li et al. [9] who reported the optical absorption peak at 3 kev from commercially available AgNPs, EDS analysis gives the additional evidence for the reduction of AgNPs; the optical absorption peak at 3 kev reported by Parashar et al. [17] is typical for the absorption of metallic AgNPs due to surface Plasmon resonance, which confirms the presence of nanocrystalline elemental silver (see Figure 6).

### 3.2. Bactericidal Activities of AgNPs

**Figure 7:**
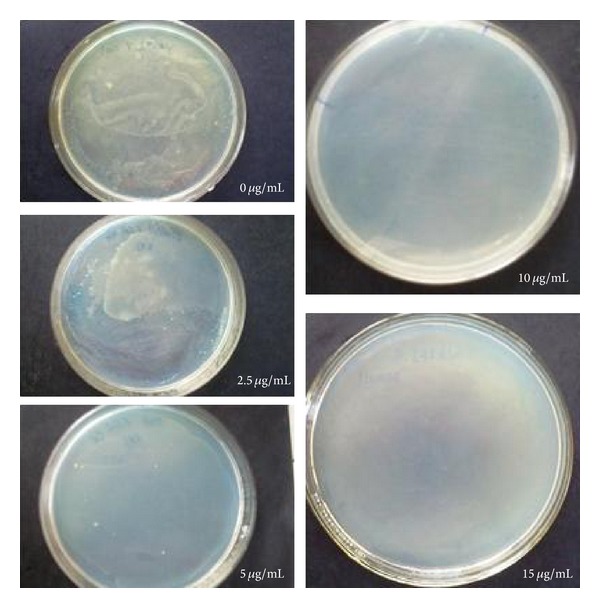
Bactericidal activities of AgNPs on* E. coli *at different concentrations ranging from 0 *μ*g/mL to 15 *μ*g/mL.

The bactericidal activity was performed against Gram negative* E. coli* on NA plates containing different concentrations of AgNPs such as 0, 2.5, 5, 10, and 15 *μ*g/mL (Figure 7). Number of bacterial colonies grew on NA plate as a function of concentration of AgNPs when overnight* E. coli* culture was applied to the plates. The presence of AgNPs at concentrations of 10 and 15 *μ*g/mL inhibited bacterial growth by 100%. There was luxurious growth at 0 *μ*g/mL, that is, without AgNPs while at 2.5 *μ*g/mL of AgNPs there was substantial growth, maybe because the concentration of 2.5 *μ*g/mL of AgNPs is not sufficient to kill* E. coli*, while at 5 *μ*g/mL there was decreased growth due to enhanced AgNPs concentration. Our results indicate that 10 *μ*g/mL of AgNPs are sufficient to inhibit 10^7^ CFU/mL of* E. coli*. For confirmation we went for 15 *μ*g/mL concentration of AgNPs against* E. coli.* Here also there was complete inhibition that indicates that a concentration of 10 *μ*g/mL of AgNPs is sufficient to completely kill 10^7^ CFU/mL of* E. coli*.

Li et al. [9] also reported that 10 *μ*g/mL of AgNPs are sufficient to completely inhibit 10^7^ CFU/mL of* E. coli*; though the AgNPs were not biologically synthesized, but purchased commercially, yet our results correlate with them. Sondi and Salopek-Sondi [13] also reported that a concentration of 20 *μ*g/mL cm^−3^ completely prevented bacterial growth if 10^4^ CFU of* E. coli* were used. While Kim et al. [14] reported a MIC of 100 *μ*g/mL of AgNPs to cause complete death of* S. aureus* and* E. coli*. Comparing our results with the above said by authors, AgNPs produced by* A. niger* are more effective in completely inhibiting* E. coli* at the minimum dose of 10 *μ*g/mL. Hiremath et al. [19] also reported antibacterial activity of AgNPs against MDR* E. coli* strains using the fungus* Rhizopus* sp.

### 3.3. Growth Curves of Bacterial Cells Treated with AgNPs

**Figure 8:**
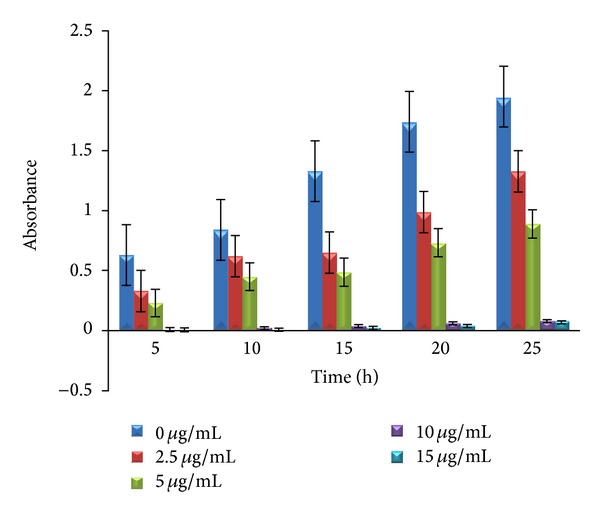
Growth curve of* E. coli* treated with AgNPs, at 0 *μ*g/mL maximum growth, at 2.5 *μ*g/mL growth reduced, at 5 *μ*g/mL bacterial growth still reduced, and at 10 and 15 *μ*g/mL growth curves diminished showing nil growth.

The growth curve of* E. coli* treated with AgNPs was determined by using NB supplemented with 0, 2.5, 5, 10, and 15 *μ*g/mL of AgNPs. The concentrations of 2.5 and 5 *μ*g/mL showed the growth of* E. coli* but comparatively less than the growth curve shown without AgNPs (0 *μ*g/mL). It is very interesting to observe that the incorporations of 10 and 15 *μ*g/mL AgNPs to NB showed complete inhibition of* E. coli* which is evident from graph in Figure 8. The dynamics of bacterial growth was monitored in liquid LB broth supplemented with 10^7^ *E. coli* cells with 10, 50, and 100 *μ*g cm^−3^ of AgNPs at all these concentrations caused a growth delay of* E. coli*. Zhou et al. [20] also reported that a concentration of 10 *μ*g/mL of AgNPs showed strong antibacterial activity against* E. coli*.

### 3.4. Release of ROS from the Surface of AgNPs and Its Mechanism

**Figure 9:**
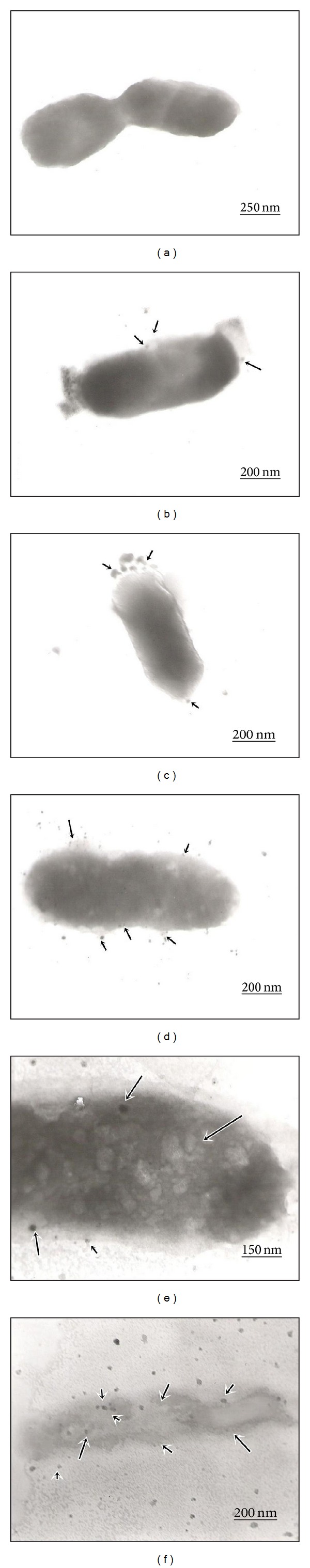
TEM micrograph of* E. coli* loaded with AgNPs. (a) Normal* E. coli* cell with its well-integrated cell wall. (b) Anchoring of AgNPs on cell wall of* E. coli*. (c) Ruptured cell membrane and entry of AgNPs into the cytoplasm. (d) Complete damage of cell wall and cell membrane. (e) Clear AgNPs within the* E. coli* cell. (f) Complete destruction of* E. coli* cell and overloading of AgNPs.

Gram negative,* E. coli* was selected as a model to study the effect of ROS released from the surface of AgNPs on the permeability and the membrane structure of* E. coli* cells. The interaction of AgNPs with bacterium was analyzed using TEM micrographs.* E. coli* samples were incubated with AgNPs for 12 hours and were analyzed by employing TEM.  Figure 9(a) shows normal* E. coli *cells with its well-integrated cell wall. When the* E. coli* cells were made to interact with AgNPs for 1 hr, it can be seen from Figure 9(b) that the AgNPs were trying to adhere to the surface of* E. coli* cells. After 5 hrs of interaction a closure look at the bacterial cell membrane reveals that AgNPs anchored onto the cell surface of* E. coli* and many pits and gaps appeared in the micrograph and their membrane was fragmented (Figure 9(c)).  Figure 9(d) shows that AgNPs are trying to get inside the bacterial cell through the ruptured cell membrane. After 8 hours, the AgNPs surround the bacterial cell surface and almost cover the sides of the cell and nanoparticles are visible in the cytoplasm also. In addition electron dense particles or precipitates were also observed around the damaged bacterial cell (Figure 9(e)). The interaction can either completely disintegrates the cell or may cause cell lyses after 12 hours; Figure 9(f) shows AgNPs eventually leading to the bacterial cell death.

Another proposed mechanism of* E. coli* membrane damage by AgNPs relates to metal depletion, that is, the formation of pits in the outer membrane and change in membrane permeability by the progressive release of lipopolysaccharide (LPS) molecules and membrane proteins. A bacterial membrane with this morphology exhibits a significant increase in permeability, leaving the bacterial cells incapable of properly regulating transport through the plasma membrane and, finally, causing cell death. It is well known that the outer membrane of* E. coli *cells is predominantly constructed from tightly packed LPS molecules, which provide an effective permeability barrier [13]. It has been also proposed that the sites of interaction for AgNPs and membrane cells might be due to sulfur containing proteins in a similar way as silver interacts with thiol groups of respiratory chain proteins and transport proteins interfering with their proper function [21–25].

**Figure 10:**
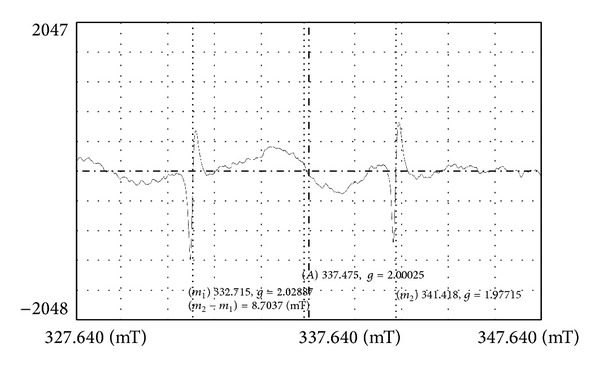
ESR spectrum of AgNPs recorded at room temperature, *m*_1_ (332.715) and *m*_2_ (341.418) indicates the control peak of Mn and the peak (mT: 337.475) indicates the released free radical from AgNPs. Instrument setting: JEOL-JES-TE 200 spectrophotometer micropower, 4 mW, MOD, 100 khz, and the time const 0.03 sec.

**Figure 11:**
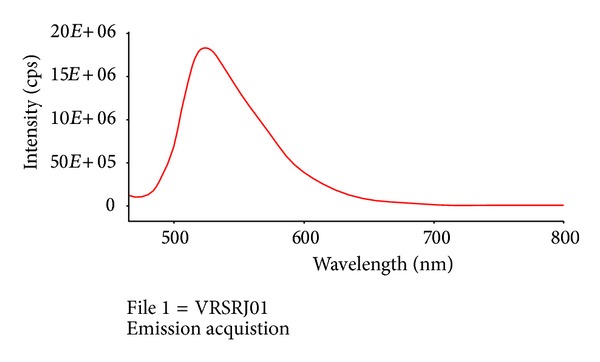
Formation of ROS in* E. coli*, using fluorescence spectroscopy.

Reactive Oxygen Species (ROS) are natural byproducts of the metabolism of respiring organisms [16]. Induction of ROS synthesis leads to the formation of highly reactive radicals that destroy the cells. The bacterial growth inhibition by formation of free radicals from the surface of AgNPs was also observed by employing ESR spectroscopy (Figure 10). Excess generation of reactive oxygen species can attack membrane lipids and then lead to a breakdown of membrane function. Certain transition metals might disrupt the cellular donor ligands that coordinate Fe. Mounting evidence suggests that the primary targets for various metals are the solvent exposed [4Fe-4S] clusters of proteins. The direct or indirect destruction of [4Fe-4S] clusters by metals could result in the release of additional Fenton-active Fe into the cytoplasm resulting in increased ROS formation. The ability to induce Fe release from these proteins, as well as from other Fe-containing proteins, might account for observations that some Fenton-inactive metals (such as Ag, Hg, and Ga) generate ROS and that cells require or upregulate ROS-detoxification enzymes to withstand toxic doses of these nanoparticles [26]. The ROS production from the surface of AgNPs was measured using DCFH-DA method. Dichlorofluorescein diacetate (DCFDA) is a popular fluorescence-based probe for reactive oxygen species (ROS) detection* in vitro*. After 5 h of incubation, ROS formed in the sample was detected at 523 nm of emission wavelength using Fluorescence spectroscopy (Figure 11). Intracellular esterases cleave DCFH-DA at the two ester bonds producing a relatively polar and cell membrane-impermeable product, H2DCF. This nonfluorescent molecule accumulates intracellularly and subsequent oxidation yields the highly fluorescent product DCF. The redox state of the sample can be monitored by detecting the increase in fluorescence. The result confirms the generation of free radicals and from the surface of AgNPs, which becomes toxic to bacterial cells leading to death. Our result corroborates with Kim et al. [14] who reported the oxidative stress can cause damage to bacterial cell membrane, protein structure, and intracelluluar system against* S. aureus* and* E. coli* using DCFDA method.

**Figure 12:**
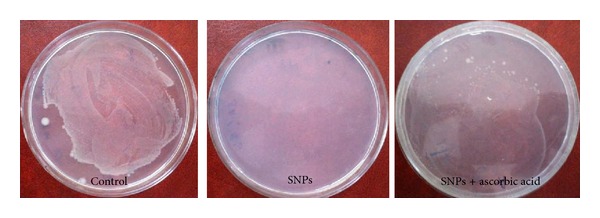
Antioxidant activities of silver nanoparticles on* E. coli.*

To determine the involvement of ROS in the antibacterial activity of AgNPs, we used ascorbic acid as scavenger. This antioxidant was used to scavenge the ROS produced by the AgNPs. Protective activity of antioxidant against bactericidal activity of AgNPs was observed in Figure 12. In control plate, the bacterial colonies were clearly seen without antioxidants and AgNPs, but in the plate supplemented with AgNPs (10 *μ*g/mL), no bacterial growth was observed revealing that AgNPs completely inhibited bacterial growth due to ROS formation. Surprisingly the bacterial colonies were observed in the plate supplemented with both AgNPs and antioxidant, which clearly indicates that the ascorbic acid used as an antioxidant serves as a scavenger hindering the ROS release from AgNPs. It is determined that the antioxidant prevents the formation of a silver oxide layer on the AgNPs surface and consequently formation of the Ag^+^ reservoir. Kora and Arunachalam [15] reported the 100%* P. aeruginosa* growth survival by using ascorbic acid as scavenger while 73% of bacteria protected from NAC as scavenger.

Bacterial cells exposed to AgNPs suffer morphological changes such as cytoplasm shrinkage, detachment of cell wall membrane, DNA condensation and localization in an electron-light region in the centre of the cell, and cell membrane degradation allowing the leakage of intracellular contents [14, 27]. Physiological changes occur together with the morphological changes; bacterial cells enter an active but nonculturable state in which physiological levels can be measured but cells are not able to grow and replicate [9, 28].

## 4. Conclusion

From our results two aspects can be elucidated, one is the fact that AgNPs themselves having antibacterial properties act on the pathogenic bacteria by anchoring to the cell surface trying to get inside the bacterial cell through ruptured cell membrane leading to the perforation of cell membrane by causing leakage of cell components and finally cell death. The second aspect is the release of ROS from AgNPs produced by* A. niger*, which is clearly evident from the facts that the antioxidant ascorbic acid is used as a scavenger. ROS production was confirmed by using DCFH-DA method using fluorescence spectroscopy. Antioxidant will inhibit ROS production thereby luxurious growth of* E. coli* was observed. Nutrient agar plate inoculated with* E. coli* supplemented with AgNPs without addition of antioxidant showed complete inhibition of* E. coli* suggesting that ROS is released from AgNPs which completely inhibit* E. coli*. Our studies suggest that 10 *μ*g/mL of AgNPs are sufficient for complete inhibition of* E. coli *which is a boon to biotechnologists to go deep for mechanism studies.
